# Preoperative Prediction of Lymph Node Metastasis in Patients With Early-T-Stage Non-small Cell Lung Cancer by Machine Learning Algorithms

**DOI:** 10.3389/fonc.2020.00743

**Published:** 2020-05-13

**Authors:** Yijun Wu, Jianghao Liu, Chang Han, Xinyu Liu, Yuming Chong, Zhile Wang, Liang Gong, Jiaqi Zhang, Xuehan Gao, Chao Guo, Naixin Liang, Shanqing Li

**Affiliations:** ^1^Department of Thoracic Surgery, Peking Union Medical College Hospital, Chinese Academy of Medical Sciences and Peking Union Medical College, Beijing, China; ^2^Peking Union Medical College, Eight-year MD Program, Chinese Academy of Medical Sciences, Beijing, China; ^3^Department of Radiology, Peking Union Medical College Hospital, Chinese Academy of Medical Sciences and Peking Union Medical College, Beijing, China

**Keywords:** non-small cell lung cancer, machine learning, lymph node metastasis, predictive model, cross-validation

## Abstract

**Background:** Lymph node metastasis (LNM) is difficult to precisely predict before surgery in patients with early-T-stage non-small cell lung cancer (NSCLC). This study aimed to develop machine learning (ML)-based predictive models for LNM.

**Methods:** Clinical characteristics and imaging features were retrospectively collected from 1,102 NSCLC ≤ 2 cm patients. A total of 23 variables were included to develop predictive models for LNM by multiple ML algorithms. The models were evaluated by the receiver operating characteristic (ROC) curve for predictive performance and decision curve analysis (DCA) for clinical values. A feature selection approach was used to identify optimal predictive factors.

**Results:** The areas under the ROC curve (AUCs) of the 8 models ranged from 0.784 to 0.899. Some ML-based models performed better than models using conventional statistical methods in both ROC curves and decision curves. The random forest classifier (RFC) model with 9 variables introduced was identified as the best predictive model. The feature selection indicated the top five predictors were tumor size, imaging density, carcinoembryonic antigen (CEA), maximal standardized uptake value (SUV_max_), and age.

**Conclusions:** By incorporating clinical characteristics and radiographical features, it is feasible to develop ML-based models for the preoperative prediction of LNM in early-T-stage NSCLC, and the RFC model performed best.

## Introduction

Lung cancer remains the leading cause of global cancer death (1). Early-T-stage non-small cell lung cancer (NSCLC) has been detected more frequently following the rapid development and employment of radiographical technology (2). An accurate nodal stage is critical for treatment decision-making (3). Currently, there are several evaluation methods, such as computed tomography (CT), positron emission tomography/CT (PET/CT), mediastinoscopy and endobronchial ultrasound transbronchial needle aspiration (EBUS-TBNA), that can be used to classify the nodal stage before operation. However, performing mediastinoscopy or EBUS-TBNA is not cost-effective for patients with early-stage NSCLC. Furthermore, although CT and PET/CT have been widely used for the preoperative evaluation of lung cancer, the incidence of occult lymph node metastasis (LNM) in early-T-stage NSCLC remains high and cannot be ignored (4, 5). Therefore, new reliable methods for the preoperative prediction of LNM are highly required.

Machine learning (ML) is an emerging computer-based method that has been widely used for data analysis in medicine during the past decade (6, 7). It learns from data and finds the dataset pattern to identify the outcome (7, 8). Supervised ML is a process in which the model is trained with fully labeled and classified data. Compared with conventional statistical methods such as logistic regression (LR), which relies on predetermined models, ML can deeply detect the interactions among variations and iteratively learn from data to update algorithms (9).

A number of predictive models have been made based on ML algorithms. Several studies have reported effective ML-based models for the prediction of LNM in other carcinomas, such as breast cancer (10, 11). It was reported that radiomics could be used to predict LNM by analyzing radiological images in NSCLC (12). However, few reports have incorporated clinical characteristics and radiographical features as in our study. This study aimed to develop and validate effective ML-based models for the prediction of LNM in patients with early-T-stage NSCLC.

## Materials and Methods

### Study Population

Between January 2013 and June 2019, 1,102 patients who underwent surgical resection for NSCLC at Peking Union Medical College Hospital were included in this study. The inclusion criteria were as follows: (1) single NSCLC lesion; (2) tumor maximum diameter ≤ 2 cm on CT; and (3) receiving lung resection with systematic lymph node dissection. The exclusion criteria were as follows: (1) small cell lung cancer (SCLC); (2) multiple lung cancer; (3) receiving radiotherapy or chemotherapy before surgery; (4) distant metastasis; and (5) incomplete clinical records. The pathological classification of carcinomas was based on the 2015 World Health Organization (WHO) classification (13). The clinical and pathological staging was performed according to the 8th edition of the TNM staging system (14). This study was approved by the Ethics Committee of Peking Union Medical College Hospital. All patients signed informed consent before operation.

### Clinical Characteristics and Radiographical Features

A total of 23 variables were analyzed in this study. The patients' clinical characteristics included age, sex, smoking status and serum tumor biomarkers. All preoperative serum tumor biomarkers were measured within 3 months before surgery, including carbohydrate antigen 24-2 (CA242), squamous cell carcinoma antigen (SCCAg), carcinoembryonic antigen (CEA), carbohydrate antigen 19-9 (CA199), carbohydrate antigen 12-5 (CA125), carbohydrate antigen 72-4 (CA724), carbohydrate antigen 15-3 (CA153), neuron-specific enolase (NSE), tissue polypeptide-specific antigen (TPS), cytokeratin 19-fragments (Cyfra211) and pro-gastrin-releasing peptide (proGRP). CT features were reviewed by one radiologist and two thoracic surgeons independently, including tumor location side, tumor maximum size, spiculation, vessel convergence, lobulation, pleural indentation, calcification, and imaging density. If disagreement occurred, the final result was reached by consensus. Based on imaging density on CT, the cancer lesions were divided into pure ground-glass opacity (pGGO), mixed GGO (mGGO) and solid nodules. The mGGO was further divided into two groups according to different percentages of solid components, whose cut-off value was 50% (the ratio between the maximal diameter of the solid component at the mediastinal window and the maximal tumor diameter at the lung window). In addition, the maximal standardized uptake value (SUV_max_) on PET/CT was also included. However, PET scan was not routinely performed in early-T-stage NSCLC. All patients underwent CT or PET scan within 60 days at our hospital before the operation.

### Construction of ML-Based Models

All patients were randomly divided into training and testing groups at a ratio of 8:2, keeping the distribution of node-positive and node-negative data in both groups consistent. To construct more reliable ML-based predictive models, all continuous variables were preprocessed by z-score normalization except for multinomial naïve Bayes (MNB) in which min-max normalization is preferred (15). Some continuous variables with missing data (Table S1), such as SUV_max_ and tumor biomarkers, were imputed by median value (16, 17).

Eight algorithms were applied to predict LNM, including adaptive boosting (AdaBoost), artificial neural network (ANN), decision tree (DT), gradient boosting decision tree (GBDT), logistic regression (LR), MNB, random forest classifier (RFC), and extreme gradient boosting (XGBoost) (18–23). Among all 8 algorithms, LR and MNB are considered conventional methods, and the others are representative supervised ML-based algorithms. Only DT, LR, and MNB were interpretable, in which users were able to recognize function between variable and predictive outcome.

The prediction ability of the 8 models was first evaluated by the receiver operating characteristic (ROC) curve, which is a conventional diagnostic test method that only pays attention to the sensitivity and specificity but ignores the clinical utility of predictive information. Decision curve analysis (DCA) was performed to calculate the clinical values of these models, which is a novel method to assess the information value between diagnostic models by considering the possible range of a patient's risk and benefit preferences without actually measuring these preferences for one particular patient (24).

### Validation Strategy and Feature Selection

Overfitting is a common problem in ML, especially with high dimensions (number of variables). To minimize the negative influence of overfitting, some strategies, such as the preselection of variables and cross-validation, were feasible (25, 26). Therefore, 5-fold cross-validation and feature selection were performed in this study. The 5-fold cross-validation randomly split the dataset into 5 subsets. For each repeated time, four subsets were used as the training group and the remaining subset was used as the testing data. This procedure was repeated 5 times, and each subset should be used exactly once as the testing group. To rank and select meaningful variables, a classifier-specific evaluator was used, returning a ranked list of variables for each algorithm. The ranks of each variable in different algorithms were compared, and the variables with high ranks were identified.

### Statistical Analysis

Univariate analysis was performed using IBM SPSS 25.0 (SPSS Inc; Chicago, IL, USA). Quantitative data were first tested for normality by the Shapiro-Wilk test. Normal data are expressed as the mean ± standard deviation (SD), while non-normal data are expressed as the median with interquartile range (IQR). Student's *t*-test was used to compare normal quantitative parameters, while the Mann-Whitney U test was used to compare non-normal quantitative parameters. For categorical data, Pearson's chi square test or Fisher's exact test was applied. Python programming language (version 3.7, Python Software Foundation) was used for the construction of ML models and DCA. Student's *t*-test was also used for the comparison of different ML models (AUCs). A *P*-value < 0.05 was considered statistically significant.

## Results

### Patient Characteristics

All 1,102 patients' clinical characteristics and radiographical features are listed in Table 1. Univariate analysis was performed for data without a median value imputed. LNM occurred in 10.5% (116/1102) of patients with NSCLC ≤ 2 cm. In total, 699 (63.4%) patients were female, and LNM occurred more frequently in smokers (*P* = 0.026). The maximum tumor size on CT in patients with positive nodes was significantly larger than that in patients with negative nodes (*P* < 0.001). All patients had a maximal diameter no smaller than 4 mm. Tumor imaging density (*P* < 0.001) and pleural indentation (*P* = 0.006) also presented significant differences between node-positive and node-negative patients. None of the patients with positive nodes in this study had a pGGO cancer nodule. Moreover, patients with LNM were significantly different from those without LNM in 4 serum tumor biomarkers: CEA (*P* < 0.001), CA125 (*P* = 0.001), CA153 (*P* = 0.030), and Cyfra211 (*P* = 0.013).

**Table 1:** Univariate analysis of patients' clinical characteristics and image features.

	**Total**	**Lymph node status**		***P*-value**
		**pN_**+**_**	**pN_**0**_**	
All patients	1102	116 (10.5)	986 (89.3)	
Age, years	58 [51–65]	59 [53–66]	58 [50–64]	0.382
Sex				
Male	403 (36.6)	52 (44.8)	351 (35.6)	0.051
Female	699 (63.4)	64 (55.2)	635 (64.4)	
Smoking status				
Yes	218 (19.8)	32 (27.6)	186 (18.9)	0.026
No	884 (80.2)	84 (72.4)	800 (81.1)	
Tumor side				
Left	461 (41.8)	49 (42.2)	412 (41.8)	0.925
Right	641 (58.2)	67 (57.8)	574 (58.2)	
Tumor size, cm	1.3 [1.0–1.7]	1.7 [1.5–2.0]	1.2 [1.0–1.6]	<0.001
Imaging density				
pGGO	431 (39.1)	0 (0.0)	431 (43.7)	<0.001
mGGO (solid <50%)	330 (30.0)	51 (44.0)	279 (28.3)	
mGGO (solid ≥ 50%)	146 (13.2)	27 (23.3)	119 (12.1)	
Solid nodule	195 (17.7)	38 (32.7)	157 (15.9)	
Spiculation				
Yes	587 (53.3)	70 (60.3)	517 (52.4)	0.106
No	515 (46.7)	46 (39.7)	469 (47.6)	
Vessel convergence				
Yes	234 (21.2)	17 (14.7)	217 (22.0)	0.067
No	868 (78.8)	99 (85.3)	769 (78.0)	
Lobulation				
Yes	403 (36.6)	52 (44.8)	351 (35.6)	0.071
No	699 (63.5)	64 (55.2)	635 (64.4)	
Pleural indentation				
Yes	294 (26.7)	43 (37.1)	251 (25.5)	0.007
No	808 (73.3)	73 (62.9)	735 (74.5)	
Calcification				
Yes	21 (1.9)	4 (3.4)	17 (1.8)	0.414
No	1081 (98.1)	112 (96.6)	969 (98.2)	
Tumor SUV_max_	1.3 [0.7–2.9]	5.9 [3.2–8.7]	1.2 [0.7–2.3]	<0.001
CA242	6.4 [3.4–12.7]	7.5 [4.5–16.5]	6.1 [3.3–12.5]	0.131
SCCAg	0.8 [0.6–1.0]	0.8 [0.6–1.0]	0.8 [0.6–1.0]	0.473
CEA	1.89 [1.20–2.83]	3.63 [2.08–6.69]	1.79 [1.15–2.60]	<0.001
CA199	10 [6.8–16.9]	12.1 [7.4–22.0]	9.9 [6.7–16.8]	0.072
CA125	10.7 [8.0–15.0]	13.3 [9.0–30.1]	10.5 [7.9–14.1]	0.001
CA724	1.9 [1.2–4.3]	2.5 [1.4–5.6]	1.9 [1.2–4.2]	0.128
CA153	9.6 [7.3–13.1]	10.6 [8.0–14.4]	9.5 [7.2–12.9]	0.030
NSE	13.6 [11.6–15.6]	13.5 [11.8–15.8]	13.6 [11.5–15.6]	0.577
TPS	46.68 [29.41–83.10]	54.22 [28.77–110.40]	46.68 [29.30–79.80]	0.492
Cyfra211	1.92 [1.42–2.68]	2.01 [1.63–2.97]	1.90 [1.40–2.62]	0.013
ProGRP	32.1 [26.0–40.5]	33.6 [26.5–45.4]	32.1 [26.0–40.1]	0.115

*pGGO, pure ground glass opacity; mGGO, mixed ground glass opacity; Solid <50%/Solid > 50%: the ratio between the maximal diameter of the solid component at the mediastinal window and the maximal tumor diameter at the lung window <50%/ > 50% in mGGO; SUV_max_, maximal standardized uptake value; CA242, carbohydrate antigen 24-2; SCCAg, squamous cell carcinoma antigen; CEA, carcinoembryonic antigen; CA199, carbohydrate antigen 19-9; CA125, carbohydrate antigen 12-5; CA724, carbohydrate antigen 72-4; CA153, carbohydrate antigen 15-3; NSE, neuron-specific enolase; TPS, tissue polypeptide-specific antigen; Cyfra211, cytokeratin 19-fragments; proGRP, pro-gastrin-releasing peptide*.

### Predictive Performance and Clinical Utility of ML-Based Models

**Figure 1:**
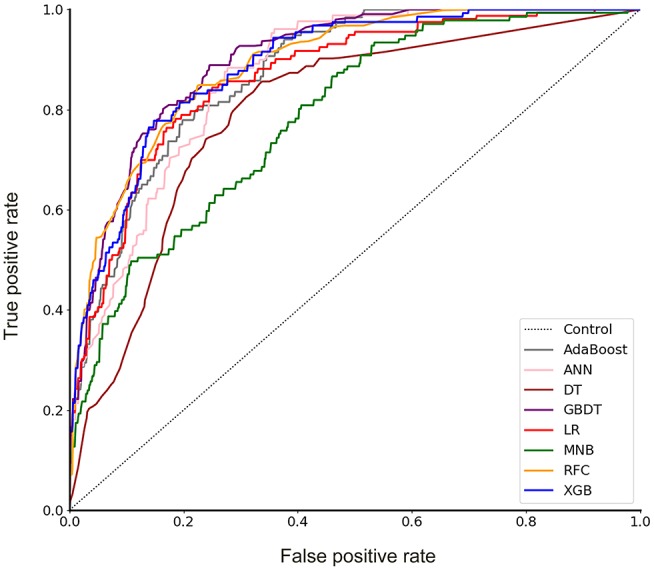
Receiver operating characteristic (ROC) curve for 8 models. AdaBoost, adaptive boosting; ANN, artificial neural network; DT, decision tree; GBDT, gradient boosting decision tree; LR, logistic regression; MNB, multinomial naïve Bayes; RFC, random forest classifier; XGBoost, extreme gradient boosting.

A total of 23 preoperative variables were used to develop predictive models for LNM based on 8 algorithms. The predictive performance of all models is shown in Figure 1 and Table 2. The best performance was observed in the GBDT model (AUC = 0.899, *SD* = 0.048), which performed similarly to RFC (AUC = 0.890, *SD* = 0.045, *P* = 0.773), XGBoost (AUC = 0.883, *SD* = 0.047, *P* = 0.627), AdaBoost (AUC = 0.873, *SD* = 0.048, *P* = 0.432), and ANN (AUC = 0.868, *SD* = 0.049, *P* =0.341). All ML-based models except DT (AUC = 0.802, *SD* = 0.057) were better than the two conventional methods, LR (AUC = 0.867, *SD* = 0.049, *P* = 0.338) and MNB (AUC = 0.784, *SD* = 0.058, *P* = 0.002). Moreover, all models performed significantly better than using only tumor size (AUC = 0.753, *SD* = 0.023, *P* < 0.001; the cut-off value was 1.5 cm), SUV_max_ (AUC = 0.734, *SD* = 0.024, *P* < 0.001; the cut-off value was 2.8) or CEA (AUC = 0.720, *SD* = 0.026, *p* < 0.001; the cut-off value was 2.98 ng/ml).

**Table 2:** Predictive performance (AUC) of 8 models and using several variables alone.

**Model**	**AUC**			**No. of optimal dimensions**
	**Mean**	**SD**	**95% CI**	
AdaBoost	0.873	0.048	0.779–0.968	7
ANN	0.868	0.049	0.772–0.964	7
DT	0.802	0.057	0.691–0.913	2
GBDT	0.899	0.044	0.813–0.985	11
LR	0.867	0.049	0.771–0.963	13
MNB	0.784	0.058	0.670–0.898	11
RFC	0.890	0.045	0.801–0.979	13
XGBoost	0.883	0.047	0.792–0.975	7
Tumor size	0.753	0.023	0.707–0.798	1
SUV_max_	0.734	0.024	0.688–0.780	1
CEA	0.720	0.026	0.669–0.770	1

*AUC, area under the receiver operating characteristic curve; AdaBoost, adaptive boosting; ANN, artificial neural network; DT, decision tree; GBDT, gradient boosting decision tree; LR, logistic regression; MNB, multinomial naïve Bayes; RFC, random forest classifier; XGBoost, extreme gradient boosting; SUV_max_, maximal standardized uptake value; CEA, carcinoembryonic antigen; CA125, carbohydrate antigen 12-5; Cyfra211, cytokeratin 19-fragments; CA153, carbohydrate antigen 15-3*.

**Figure 2:**
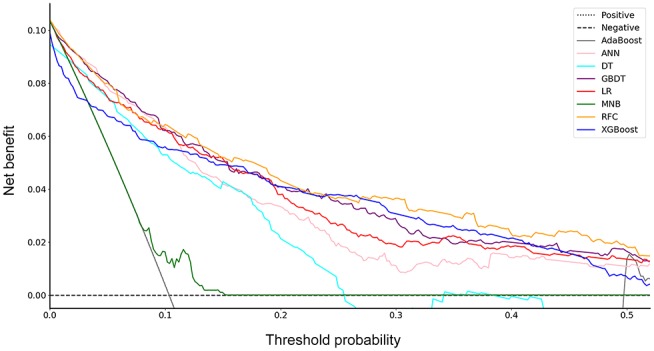
Decision curve for 8 models. AdaBoost, adaptive boosting; ANN, artificial neural network; DT, decision tree; GBDT, gradient boosting decision tree; LR, logistic regression; MNB, multinomial naïve Bayes; RFC, random forest classifier; XGBoost, extreme gradient boosting.

Furthermore, the decision curve showed the clinical values of these models (Figure 2). The net benefits of 8 models at each threshold probability are shown in Table S2. Most of these models presented better net benefits than two control models that were represented by positive and negative line, respectively. The negative line represents the net benefit is zero when none of patients receive lobectomy with systematic lymph node dissection (SND), assuming that all patients have no positive nodes. On the contrary, the positive line represents the net benefits at the time when all patients have positive nodes and receive lobectomy with SND. Four models (RFC, XGBoost, GBDT, and LR) performed significantly better than the others at most of threshold points. At the range of 0.2–0.5, the LR model was less beneficial than RFC, XGBoost and GBDT on most occasions. The RFC model with 9 variables introduced, which achieved a very high AUC (0.890) and had the highest net benefits almost across the entire range of threshold probabilities, was regarded as the best predictive model in this study, although its AUC value was slightly lower than that of GBDT (*P* = 0.773).

### Variable Importance

**Figure 3:**
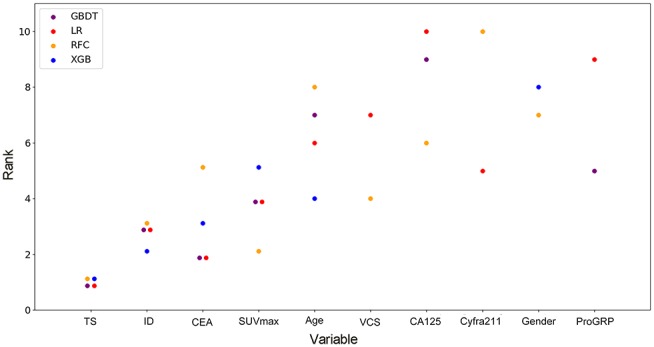
Ranks of the top 10 variables for the prediction of lymph node metastasis. Variables were ranked using a classifier-specific evaluator based on machine learning algorithms. Each variable was ordered according to their mean ranks. The lower rank represents more contributions to the prediction of lymph node metastasis. For example, SUV_max_ was ranked 2nd, 3rd, 3^rd^, and 5th in RFC, GBDT, LR, and XGB, respectively. TS, tumor size; ID, imaging density; CEA, carcinoembryonic antigen; SUVmax, maximal standardized uptake value; VCS, vessel convergence sign on CT; CA125, carbohydrate antigen 12-5; Cyfra211, cytokeratin 19-fragments; proGRP, pro-gastrin-releasing peptide.

**Figure 4:**
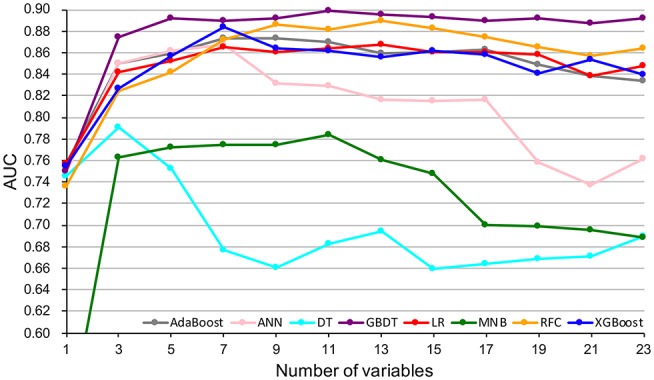
Predictive performance (AUCs) of 8 models as number of variables increases. AdaBoost, adaptive boosting; ANN, artificial neural network; DT, decision tree; GBDT, gradient boosting decision tree; LR, logistic regression; MNB, multinomial naïve Bayes; RFC, random forest classifier; XGBoost, extreme gradient boosting.

**Figure 5:**
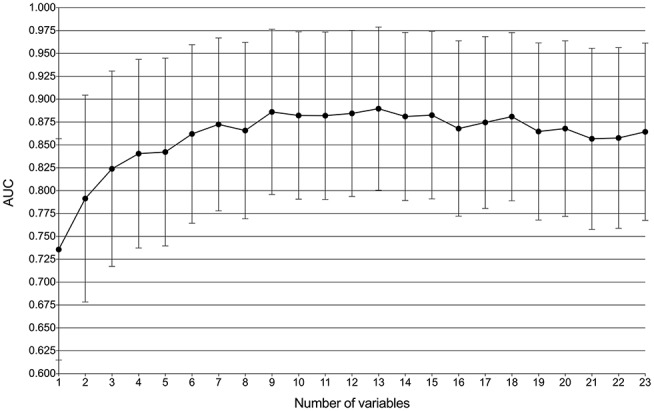
Predictive performance (AUCs) of the random forest classifier (RFC) model at each number of variables.

By feature selection, the 23 variables for each algorithm were ranked by their predictive importance (Table S3). The top 10 variables are shown in Figure 3. The five top-ranked predictors were tumor size, imaging density, CEA, SUV_max_, and age. The relationship between the AUCs of models and the number of variables were evaluated in Figure 4. The AUCs of most models reached a plateau when 7 variables were introduced, while those of ANN, DT, and MNB started to drop down when they reached the highest points. The AUCs of RFC for each number of variables are shown in Figure 5. Its AUC value reached a plateau when 9 variables were introduced and reached the highest value when 13 variables were introduced, but it did not increase significantly with the change from 9 variables (AUC = 0.886) to 13 variables (AUC = 0.890) introduced. Considering the clinical utility, the 9 top-ranked variables were identified to construct the optimal predictive model, which included tumor size, SUV_max_, imaging density, vessel convergence sign, CEA, CA125, sex, age, and spiculation sign.

## Discussion

Lobectomy with systematic lymph node dissection remains the standard treatment for patients with early-T-stage NSCLC (≤ 2 cm) (27). However, sublobar resection, including segmentectomy and wedge resection, has been proposed to achieve more precise intervention with the advancement of imaging techniques in recent years. In addition, the reasonable extent of lymph node dissection remains controversial. An exact nodal status is critical for treatment selection and prognosis.

In this study, using ML algorithms, we developed 8 models to predict LNM in 1,102 patients with NSCLC ≤ 2 cm, incorporating their clinical characteristics and radiographical features. ROC analysis and DCA were used to evaluate the predictive performance and clinical values of the models, respectively. Most of 8 models maintained high AUCs and All ML-based models (with AUCs ranging from 0.868 to 0.899) except DT performed better than two models using conventional statistical methods (LR and MNB) in the prediction of LNM (Figure 1 and Table 2).

DCA has been used for many medical studies and has shown great clinical utility (28, 29). In the decision curve, most of these models performed better than positive line and negative line, indicating that the overall net benefit of giving lobectomy with SND to patients identified by the models to have high risk of LNM was higher than that of giving the same surgical procedures to all patients or no patient. Four models (RFC, XGBoost, GBDT, and LR) performed better than the others at most of threshold points (Figure 2). Thus, these four potential models were used to identify variable importance by feature selection (Figure 3). The other four models, AdaBoost, MNB, DT, and ANN, had lower net benefits in the decision curve (Figure 2), although they possessed high AUCs in the ROC curve. This indicated that models with high predictive accuracy might not be clinically practical and require further evaluation by other methods, such as DCA.

Using conventional univariate analysis, previous studies reported the risk factors associated with LNM in NSCLC ≤ 2 cm, including tumor size, serum CEA and imaging density (30, 31). In addition, SUV_max_ was also thought to be a risk factor in patients with cT1 NSCLC (32). Thus, the AUCs when using tumor size (AUC = 0.753), SUV_max_ (AUC = 0.734), or CEA (AUC = 0.720) alone were also calculated, which were significantly lower than those of ML-based models (Table 2). Thus, previous studies might not provide precise predictive information for LNM. Reliable predictive models for LNM in patients with NSCLC are needed. To our knowledge, our study was the first to provide potential models for the prediction of LNM in patients with NSCLC by incorporating clinical characteristics and radiographical features.

Although most of the ML-based models in our study cannot demonstrate the connection between the predictive variables and the outcomes, the contribution of each variable to the models could be inferred by feature selection. Tumor size, imaging density, serum CEA, SUV_max_, and age were indicated to be the most contributive risk factors of LNM (Figure 3), which was similar to the results of univariate analysis (Table 1). Since none of the patients with pGGO NSCLC had positive nodes in our and previous studies (30, 31), it could be inferred that pGGO might be predictive of node-negative status in early-T-stage NSCLC. It was also reported that a higher serum CEA level was significantly associated with a higher incidence of LNM (31, 33). Although only 611 patients' SUV_max_ values (pN+: n = 62, pN0: n = 549; *p* > 0.05) were available because some patients did not undergo PET scans, SUV_max_ was ranked at 4 among the four potential models (Figure 3) and was ranked at 2 in the RFC model (Figure 4). Meanwhile, a high AUC (0.734) for SUV_max_ was also obtained. Above all, SUV_max_ might be one of the most important predictive factors, which was consistent with previous studies (32, 34). Surprisingly, age showed no significance in univariate analysis (*p* = 0.382) but was ranked at the top 5 (Figure 3). This might be attributed to the surprising superiority of ML-based models in data mining, which could find more relations between the variables and the outcomes than conventional methods.

According to the ROC curve (Figure 1) and decision curve (Figure 2), the RFC model with 9 variables introduced (AUC = 0.890) was identified as the optimal model. By considering the clinical utility, an application based on the RFC algorithm with 9 variables (AUC = 0.886) should be developed in the future. These 9 variables were tumor size, SUV_max_, imaging density, vessel convergence sign, CEA, CA125, sex, age, and spiculation sign. Thus, clinicians from other hospitals could benefit from our study.

In addition to the clinical values, there were several methodological indications in our study. First, although there were several studies of machine learning involving NSCLC, few of them have reported predictive models for LNM using ML algorithms by incorporating clinical characteristics and radiographical features. Most of them performed image analysis by radiographical data (12) or histological slides (35). This is the first study to predict LNM in NSCLC ≤ 2 cm, indicating the feasibility and potential of ML algorithms applied in NSCLC. More predictive models of NSCLC may be developed using ML algorithms to solve clinical problems in the future. Second, based on ROC analysis and DCA, multiple supervised ML algorithms performed better than conventional methods. Thus, the ML algorithms would play an important role in the analysis of large medical datasets. Third, in addition to the ROC curve, a decision curve was used to evaluate the clinical utility of these models. Some models performed worse in the decision curve, although they had very high AUCs. This provides a method to further evaluate the clinical values of ML-based models.

There were also some limitations in our study. First, there were some patients who received sublobar resection (wedge resection or segmentectomy), and thus, the incidence of LNM in this population might have been underestimated. Second, missing data were inevitable. This is because not all patients with early-T-stage NSCLC receive PET scans or tumor biomarker tests. Except for SUV_max_ and serum biomarkers, the clinical records of other variables were complete. The median value was imputed to solve this problem (16, 17). Third, this is a retrospective study that could not completely avoid data selection and measurement biases. More prospective studies or multicenter studies may be needed to develop predictive models in the future.

## Conclusions

ML-based models are effective in the prediction of LNM in NSCLC ≤ 2 cm by incorporating clinical and radiographical characteristics. Based on ROC analysis and DCA, some ML-based models performed better than models using conventional methods, and the RFC model performed best. The feature selection approach identified that tumor size, imaging density, CEA, SUV_max_, and age were the most important predictive risk factors for LNM.

## Data Availability Statement

All datasets generated for this study are included in the article/Supplementary Material.

## Ethics Statement

The studies involving human participants were reviewed and approved by Ethics Committee of Peking Union Medical College Hospital. Written informed consent to participate in this study was provided by the participants' legal guardian/next of kin.

## Author Contributions

SL, NL, YW, and JL: conceptualization. YW and JL: methodology. YW, JL, CH, XL, and YC: formal analysis. YW, ZW, LG, JZ, XG, and CG: investigation. YW and JL: writing—original draft preparation. YW, SL, and NL: writing—review and editing. SL: supervision.

## Conflict of Interest

The authors declare that the research was conducted in the absence of any commercial or financial relationships that could be construed as a potential conflict of interest.
