# Supplementary material for: Preoperative Prediction of Lymph Node Metastasis in Patients With Early-T-Stage Non-small Cell Lung Cancer by Machine Learning Algorithms
Source: Front Oncol. 2020 May 13;10:743. doi: 10.3389/fonc.2020.00743 (PMC7237747; doi:10.3389/fonc.2020.00743)
Supplement: Supplementary file 1 [file Table_1.DOCX]

**Table S1. Patients with available data for SUV_max_ and tumor biomarkers.**

|  | Total | lymph node status | | p value |
| --- | --- | --- | --- | --- |
|  |  | pN_+_ | pN_0_ |  |
| Tumor SUVmax |  |  |  |  |
| Avaiable | 611 | 62 | 549 | 0.647 |
| Missing | 491 | 54 | 437 |  |
| CA242 |  |  |  |  |
| Avaiable | 541 | 53 | 488 | 0.438 |
| Missing | 561 | 63 | 498 |  |
| SCCAg |  |  |  |  |
| Avaiable | 900 | 94 | 806 | 0.852 |
| Missing | 202 | 22 | 180 |  |
| CEA |  |  |  |  |
| Avaiable | 930 | 93 | 837 | 0.186 |
| Missing | 172 | 23 | 149 |  |
| CA199 |  |  |  |  |
| Avaiable | 585 | 60 | 525 | 0.756 |
| Missing | 517 | 56 | 461 |  |
| CA125 |  |  |  |  |
| Avaiable | 503 | 56 | 447 | 0.547 |
| Missing | 599 | 60 | 539 |  |
| CA724 |  |  |  |  |
| Avaiable | 495 | 45 | 450 | 0.161 |
| Missing | 607 | 71 | 536 |  |
| CA153 |  |  |  |  |
| Avaiable | 538 | 52 | 486 | 0.363 |
| Missing | 564 | 64 | 500 |  |
| NSE |  |  |  |  |
| Avaiable | 831 | 88 | 743 | 0.905 |
| Missing | 271 | 28 | 243 |  |
| TPS |  |  |  |  |
| Avaiable | 532 | 50 | 482 | 0.239 |
| Missing | 570 | 66 | 504 |  |
| Cyfra211 |  |  |  |  |
| Avaiable | 896 | 93 | 803 | 0.740 |
| Missing | 206 | 23 | 183 |  |
| ProGRP |  |  |  |  |
| Avaiable | 691 | 76 | 615 | 0.508 |
| Missing | 411 | 40 | 371 |  |

SUV_max_: maximal standardized uptake value; CA242: carbohydrate antigen 24-2; SCCAg: squamous cell carcinoma antigen; CEA: carcinoembryonic antigen; CA199: carbohydrate antigen 19-9; CA125: carbohydrate antigen 12-5; CA724: carbohydrate antigen 72-4; CA153: carbohydrate antigen 15-3; NSE: neuron specific enolase; TPS: tissue polypeptide specific antigen; Cyfra211: cytokeratin 19-fragments; proGRP: pro-gastrin-releasing peptide.
