# Supplementary material for: Preoperative Prediction of Lymph Node Metastasis in Patients With Early-T-Stage Non-small Cell Lung Cancer by Machine Learning Algorithms
Source: Front Oncol. 2020 May 13;10:743. doi: 10.3389/fonc.2020.00743 (PMC7237747; doi:10.3389/fonc.2020.00743)
Supplement: Supplementary file 2 [file Table_2.DOCX]

**Table S2. Net benefits of the 8 models at each threshold probability in the decision curve.**

| Probability (%) | Net benefit | | | | | | | | |
| --- | --- | --- | --- | --- | --- | --- | --- | --- | --- |
|  | Positive | AdaBoost | ANN | DT | GBDT | LR | MNB | RFC | XGBoost |
| 5 | 0.0562 | 0.0562 | 0.0784 | 0.0746 | 0.0807 | 0.0747 | 0.0562 | 0.0800 | 0.0675 |
| 10 | 0.0036 | 0.0036 | 0.0633 | 0.0529 | 0.0621 | 0.0625 | 0.0160 | 0.0644 | 0.0559 |
| 15 | -0.0551 | -0.0551 | 0.0418 | 0.0428 | 0.0504 | 0.0514 | 0 | 0.0521 | 0.0492 |
| 20 | -0.1212 | -0.1212 | 0.0334 | 0.0213 | 0.0410 | 0.0381 | 0 | 0.0432 | 0.0409 |
| 25 | -0.1962 | -0.1956 | 0.0196 | 0.0028 | 0.0355 | 0.0261 | 0 | 0.0369 | 0.0379 |
| 30 | -0.2819 | -0.2787 | 0.0110 | -0.0051 | 0.0286 | 0.0190 | 0 | 0.0366 | 0.0306 |
| 35 | -0.3808 | -0.3285 | 0.0118 | 0.0003 | 0.0194 | 0.0224 | 0 | 0.0297 | 0.0262 |
| 40 | -0.4962 | -0.3163 | 0.0141 | -0.0005 | 0.0199 | 0.0180 | 0 | 0.0225 | 0.0216 |
| 45 | -0.6327 | -0.2522 | 0.0125 | -0.0077 | 0.0167 | 0.0148 | 0 | 0.0242 | 0.0158 |
| 50 | -0.7967 | 0.0135 | 0.0108 | -0.0064 | 0.0163 | 0.0135 | 0 | 0.0181 | 0.0070 |
| 60 | -1.2481 | 0.0000 | 0.0071 | -0.0014 | 0.0063 | 0.0076 | 0 | 0.0049 | -0.0017 |

AdaBoost: adaptive boosting; ANN: artificial neural network; DT: decision tree; GBDT: gradient boosting decision tree; LR: logistic regression; MNB: multinomial Naïve Bayes; RFC: random forest classifier; XGBoost: extreme gradient boosting
