# Supplementary material for: Preoperative Prediction of Lymph Node Metastasis in Patients With Early-T-Stage Non-small Cell Lung Cancer by Machine Learning Algorithms
Source: Front Oncol. 2020 May 13;10:743. doi: 10.3389/fonc.2020.00743 (PMC7237747; doi:10.3389/fonc.2020.00743)
Supplement: Supplementary file 3 [file Table_3.DOCX]

**Table S3. Ranks of 23 variables at each model.**

| Variable | ADB | ANN | DT | GBDT | LR | MNB | RFC | XGB | Highest rank | Mean rank |
| --- | --- | --- | --- | --- | --- | --- | --- | --- | --- | --- |
| Tumor size | 1 | 1 | 1 | 1 | 1 | 1 | 1 | 1 | 1 | 1.00 |
| Imaging density | 2 | 2 | 2 | 3 | 3 | 4 | 3 | 2 | 2 | 2.71 |
| CEA | 3 | 3 | 10 | 2 | 2 | 3 | 5 | 3 | 2 | 4.00 |
| SUV_max_ | 6 | 8 | 8 | 4 | 4 | 23 | 2 | 5 | 2 | 7.86 |
| Age | 4 | 22 | 14 | 7 | 6 | 2 | 8 | 4 | 2 | 9.00 |
| CA125 | 16 | 4 | 16 | 9 | 10 | 8 | 6 | 15 | 4 | 9.86 |
| Cyfra211 | 9 | 17 | 11 | 12 | 5 | 6 | 10 | 18 | 5 | 10.00 |
| Vessel convergence | 8 | 7 | 22 | 13 | 7 | 18 | 4 | 12 | 4 | 11.29 |
| CA242 | 10 | 11 | 15 | 6 | 13 | 11 | 14 | 21 | 6 | 11.43 |
| SCCAg | 11 | 9 | 18 | 16 | 8 | 9 | 11 | 14 | 8 | 11.71 |
| Gender | 12 | 12 | 5 | 20 | 14 | 17 | 7 | 8 | 5 | 12.43 |
| ProGRP | 13 | 20 | 20 | 5 | 9 | 7 | 16 | 23 | 5 | 12.86 |
| Spiculation | 5 | 13 | 6 | 17 | 21 | 22 | 9 | 16 | 5 | 13.29 |
| Lobulation | 18 | 6 | 4 | 11 | 17 | 16 | 22 | 20 | 4 | 13.43 |
| CA153 | 14 | 14 | 21 | 8 | 18 | 14 | 13 | 11 | 8 | 14.57 |
| Calcification | 20 | 5 | 3 | 22 | 20 | 15 | 19 | 6 | 3 | 14.86 |
| Tumor side | 17 | 10 | 9 | 14 | 23 | 19 | 15 | 9 | 9 | 15.29 |
| Smoking status | 22 | 15 | 7 | 10 | 15 | 21 | 20 | 22 | 7 | 15.71 |
| CA724 | 15 | 19 | 19 | 15 | 11 | 13 | 18 | 10 | 11 | 15.71 |
| TPS | 23 | 18 | 17 | 21 | 12 | 12 | 12 | 19 | 12 | 16.43 |
| Pleural indentation | 7 | 16 | 12 | 18 | 22 | 20 | 21 | 13 | 7 | 16.57 |
| CA199 | 19 | 21 | 13 | 23 | 19 | 10 | 17 | 7 | 10 | 17.43 |
| NSE | 21 | 23 | 23 | 19 | 16 | 5 | 23 | 17 | 5 | 18.57 |

AUC: area under the receiver operating characteristic curve; AdaBoost: adaptive boosting; ANN: artificial neural network; DT: decision tree; GBDT: gradient boosting decision tree; LR: logistic regression; MNB: multinomial Naïve Bayes; RFC: random forest classifier; XGBoost: extreme gradient boosting; SUV_max_: maximal standardized uptake value; CA242: carbohydrate antigen 24-2; SCCAg: squamous cell carcinoma antigen; CEA: carcinoembryonic antigen; CA199: carbohydrate antigen 19-9; CA125: carbohydrate antigen 12-5; CA724: carbohydrate antigen 72-4; CA153: carbohydrate antigen 15-3; NSE: neuron specific enolase; TPS: tissue polypeptide specific antigen; Cyfra211: cytokeratin 19-fragments; proGRP: pro-gastrin-releasing peptide.
